# Obituary

**Published:** 1920-11

**Authors:** 


### OBITUARY

### Dr. William John Younger

With sincere regret we announce the death of Dr. William J. Younger, for many years a prominent dentist of San Francisco, on July 22nd, at his Paris home.

Dr. Younger was born in Santiago, Chile, October 9, 1838, of British parents. When he was ten years of age he came to San Francisco with his parents. Left upon his own resources at a very early age, he developed indomitable will and tireless energy. He succeeded in overcoming the most serious difficulties, reaching a high place in the community and a unique station in his chosen profession.

Dr. Younger was famous internationally in dental and medical circles. He was a member of the International Medical Society, and was one of the few Americans asked to read papers before that body in its meetings in Moscow, Vienna and Paris.

On March 13, 1862, Dr. Younger received his diploma from the medical department of the University of the Pacific, and when that institution became the Cooper Medical College, he was also awarded a degree. After graduation Dr. Younger practiced general medicine for a year, but thereafter devoted his time entirely to the practice of dental surgery, specializing on the treatment of oral deformities and diseases of the mouth and teeth. It would be difficult to enumerate the services this man has rendered during a long and active professional life.

In 1877 he made transplantation of teeth a successful operation devoid of danger from infection, and in 1855 invented the operation of transplantation. Upon this subject he has given clinics in almost all the large cities of the United States and Europe. He is credited also with being the originator of the first bridgework on record, in 1872.

He was a life member, one of the founders, and former president of both the California State Dental Association and the San Francisco District Dental Society.

Throughout his professional career Dr. Younger has ever been active in the interest of dental surgery and has always taken an active part in both scientific and professional societies, where his ability and merit have ever been recognized. He was a delegate from the California State Medical Association and California State Dental Association to the meetings of the International Medical Congress at Berlin in 1890, Rome 1893, Moscow 1894, and Paris 1900.

He has written many papers, each representing original research, many of which have been published. His examination of the mummy of one of the Pharaohs furnished in part, material for an interesting paper, ''Pyorrhea Alveolaris in the Times of the Pharaohs and Present Egyptians,'' read before the American Dental Society of Europe, at Geneva, Switzerland, 1905.

In his social life, Dr. Younger's genial nature and broad culture had won for him a host of friends, who will sincerely mourn his loss. He was a member of the Pioneers' Society, the Bohemian Club, a Knight Templar of California Commandery No. 1, and also a Knight Kadosh, 32nd degree, Scottish Rite Mason.

The prominent position he had attained among the medical and dental profession will forever endure both at home and abroad.

A brother, Dr. E. A. Younger, his widow, three daughters—Miss Maude Younger, the Baroness Nugent of Vienna, and Mrs. Burns McDonald, 3466 Jackson Street—and a son, Herbert, survive him.—*Pacific Gazette.*

### Dr. Maurice J. Sullivan

Dr. Maurice J. Sullivan, one of the most widely known dentists of the Pacific Coast, died at his home, 525 Baker Street, July 13th.

Dr. Sullivan was born in Marysville, February 1, 1858. He graduated from the University of Michigan, Ann Arbor, 1880. On the organization of the dental department of the University of California he was appointed demonstrator of operative dentistry, later filling the following chairs: clinical professor of operative dentistry; dental pathology; therapeutics and materia medica.

He was a member of the first State Board of Dental Examiners and Dental Surgeons at St. Mary's Hospital, 1887-90.

For the last fifteen years he was professor of therapeutics, operative dentistry and dental pathology at the College of Physicians and Surgeons. He was prominently identified with the old Cosmos Club, and at the time of his death was a member of the Union League Club.

Dr. Sullivan is survived by a brother, William Sullivan, and by a sister, Nellie Sullivan.—*Pacific Gazette.*

---

### William G. Ebersole

Dr. Ebersole was born November 18, 1864, and died in Cleveland October 6, 1920.

The dental profession records with sincere sorrow the death of Dr. Ebersole. His life was filled with professional activities and important events of a most creditable character. He was an active practitioner for twenty-five years, in which he was intensely interested. He was also devoted to his public and professional duties. He was a frequent contributor to the literary and society work of the profession. He will probably be best known by the leading part which he took in introducing the campaign for the public work of better dentistry for poor children and oral hygiene. He was enthusiastic and devoted to this cause. It was undoubtedly the hard work he did in introducing oral hygiene through the National Dental Association that caused his physical breakdown, from which he never wholly recovered. He made an heroic fight and kept up his courage to the end with indomitable persistence. He has done his task commendably, though at great personal cost, and we shall get the benefit, may he have the honor. We shall at any rate cherish his memory and humanity will benefit. His friends will miss him and regret that he can no more co-operate in the cause which is dear to us all. He has met his obligations, and his was a noble part. We shall do ours when we keep up the effort to make the world better for oral health.

---

### SOME PRACTICAL HINTS

Warm the mouth mirror to prevent fogging.

Paint inlay wax model with alcohol when investing. It helps to prevent air bubbles.

In using chisels in cavity preparation put pellet of cotton in base of cavity; it often saves pain.

Keep some asbestos boiler covering handy for soldering when an investment is not necessary, as in a repair. It protects the teeth, by dampening it dries out quickly.

In taking impressions or X-ray pictures, nausea can be prevented frequently by painting the fauces lightly with oil of cassia.—Herbert B. Ward, in *Pacific Gazette.*

